# Abusive behaviours in relationships, need satisfaction, conflict styles and relationship satisfaction: mediation and moderation roles

**DOI:** 10.1186/s40359-023-01202-6

**Published:** 2023-05-16

**Authors:** Ahu Aricioglu, Sefa Kaya

**Affiliations:** 1 Psychological Counseling and Guidance Department, Pamukkale University, Denizli, Turkey; 2 359 Sokak no:60 Daire:15 Buca, Izmir, Turkey

**Keywords:** Emerging adults, Abusive behaviours, Need satisfaction, Conflict styles, Relationship satisfaction

## Abstract

**Background:**

The current study focuses on the mediator role of abusive behaviour in romantic relationships (ABRR) in the relationship between subordination, retreat, and relationship satisfaction and the moderation role of relatedness and autonomy in the relationships between ABRR and relationship satisfaction.

**Methods:**

333 (91 men, 242 women) Turkish emerging adults in relationships participated in this research. These participants completed a measure of abusive behaviour in romantic relationship, conflict resolution styles, relationship satisfaction and need satisfaction in romantic relationship. Models 1 and 4 of Process Hayes were used in SPSS 22 to investigate moderation and mediation roles.

**Results:**

According to the results, ABRR has a full mediator role in the relationship between subordination and relationship satisfaction and has a partial mediator role in the relationship between retreat and relationship satisfaction. Another result of the study showed that ABRR negatively affected relationship satisfaction and that relatedness and autonomy moderated this relationship. Moderator roles are strong when the level of relatedness and autonomy are high.

**Conclusions:**

In conclusion, subordination and retreat as well as ABRR are risk factors for relationship satisfaction for individuals in romantic relationships. Our results suggest that relatedness and autonomy present an adaptive approach and protection method associated with improved relationship satisfaction. Therefore, subordination, withdrawal, ABRR, autonomy, and relatedness should be considered in relationship satisfaction assessment and couple therapies.

## Background

Since human beings are social, they feel the need to establish romantic relationships throughout their lives. According to Sümer and Arıcak [1], romantic relationships are defined as the process of association that individuals choose of their free will, in which attachment, intimacy, and passion are at the forefront. Individuals establish romantic relationships to meet their needs, such as respect and belonging, and make their lives more meaningful [2]. Although romantic relationships exist in every period of life, it is a critical developmental stage, especially since individuals in late adolescence or emerging adulthood form their attitudes, habits, and beliefs about romantic relationships [3]. As a matter of fact, it has been established that healthy romantic relationships meet the need for intimacy, positively affect emerging adults’ identity development, and determine the quality of close relationships established in adulthood [4, 5]. Research indicates that happy couples who maintain healthy and stable romantic relationships are associated with satisfaction [6, 7]. Considering the role of relationship satisfaction in romantic relationships, it is thought that determining the factors that may affect relationship satisfaction will contribute to the literature.

One factor that may affect relationship satisfaction in romantic relationships may be conflict resolution styles. The way individuals deal with the conflicts they encounter and the reaction patterns they display is called conflict resolution style [8]. In the existing literature, it is observed that conflict is inevitable in relationships, but the methods of coping with such conflict are vital for the continuity and sustainability of the relationship [9, 10]. If partners use constructive and positive conflict resolution styles in times of conflict, relationship satisfaction, and stability increase. On the other hand, if destructive and negative conflict resolution styles are used, relationship satisfaction decreases [10–13]. As a result, it can be said that conflict resolution styles can be effective in increasing relationship satisfaction and maintaining romantic relationships.

Another factor that may affect relationship satisfaction is abusive behaviour in romantic relationship (ABRR). ABRR is physical, sexual, emotional, and psychological coercion that couples exert on each other to gain power and control or even harm the relationship [14]. ABRR can affect couples in many ways. ABRR is associated with decreased self-esteem, relationship satisfaction, and problem-solving skills [15–18]. In one study, it is seen that ABRR is not associated with decreased relationship satisfaction [19], while in another study, there are couples who experience relationship satisfaction despite abuse being a common occurence within their relationships [20]. Considering the inconsistency between research findings on the relationship between relationship satisfaction and ABRR, it is thought that investigating the effect of ABRR on relationship satisfaction will contribute to the literature.

Another factor that may affect relationship satisfaction is the satisfaction of psychological needs in a romantic relationship. Need satisfaction is based on the theory of self-determination, and according to the Self-Determination Theory, there are three basic needs: autonomy, competence, and relatedness [21]. Autonomy is associated with will, willingness, and self-affirmation; competence relates to feeling competent and effective in one’s actions, behaviors, or goals; and relatedness relates to a sense of belonging, mutual interest, and a sense of being in a relationship with significant others [22, 23]. According to the study by Patrick et al. [24], need satisfaction, relationship satisfaction, and relationship commitment were positively significant. On the other hand, it is seen to predict perceived conflict at a negative and significant level. Other studies in the literature have suggested that relationships that facilitate autonomy, competence, and relatedness in individuals result in increased relationship quality and subjective well-being [22, 25, 26]. In the study by Eryılmaz and Doğan [27], it was determined that need satisfaction has a mediator role in the relationship between the quality of romantic relationships and subjective well-being. As a result, it can be said that the satisfaction of autonomy, competence and relatedness needs are important factors for both a healthy, romantic relationship and subjective well-being. In addition, although it is important to meet these needs together in every culture, meeting these needs may differ from culture to culture. According to the study by Kagıtçıbası [28], it is seen that individuals in Turkey have an autonomous-related self-structure. The autonomous-related self is high on autonomy as well as relatedness. Finally, it can be said that the autonomous-related self-structure in Turkish culture may have a possible effect on this study.

With regard to the studies mentioned above, this study aims to explore the relationships between relationship satisfaction, ABRR, conflict resolution styles, and need satisfaction in romantic relationships in emerging adults. It is thought that determining the variables that may affect relationship satisfaction will be beneficial in terms of contributing to preventive mental health studies for emerging adults. In conclusion, this research sought to answer the following questions:

1. Does ABRR has a mediator role in the relationship between retreat and relationship satisfaction?
2. Does ABRR has a mediator role in the relationship between subordination and relationship satisfaction?
3. Does relatedness has a moderation role in the relationship between ABRR and relationship satisfaction?
4. Does autonomy has a moderation role in the relationship between ABRR and relationship satisfaction?

## Methods

### Participants

A total of 333 Turkish emerging adults who have a relationship participated in this study. In this study convenience sampling method was used. The sample contained 242 females and 91 males with an average age of 24.70 (SD = 3,20, range 17–30). 15.3% of the participants have master’s and PhD degrees, 68.8% have bachelor’s degrees, 15.3% have high school degrees, 0.6% have a secondary school, and were seniors. The average duration of the relationship is 24.30 months.

### Procedure

The participants were informed about the study and invited to participate. Participants were reached through a questionnaire created on Google Forms. This questionnaire; consists of an informed consent form, demographic information, Turkish version of the measurement tools used in the current study. Moreover, they were told they were not obligated to participate and could withdraw whenever they wanted.

### Measurements

***Abusive Behaviour Scale for Romantic Relationship (ABS).***

The ABS [29] is 25 items self-report measure designed to assess abusive behaviours of couples. It consists of four subscales: ‘ punishing behavior’, ‘behaviors that interfere with self-expression’, ‘exploitative behavior’, and ‘violent behavior’. All items are rated on a 5-point Likert scale from 1(it does not suit me at all) to 5 (it is always suitable for me). Cronbach’s alpha coefficient for the total scale was α = 0.92. The correlation of rank differences in terms of test-retest reliability of the scale was determined as 0.93 (p < .001). The Cronbach’s alpha coefficient for the present example is α = 0.91.

### Conflict Resolution Styles Scale for romantic Relationships (CRSS)

The CRSS [9] is 25 items self-report measure designed to assess the conflict resolution styles of couples. It consists of four subscales as ‘negative conflict resolution style’, ‘positive conflict resolution style’, ‘retreat’, and ‘subordination’. Respondents indicate a scale from 1 (strongly disagree) to 6 (strongly agree). Cronbach’s alpha coefficient for the sub-dimensions was α = 0.80 for positive conflict resolution style, α = 0.82 for negative conflict resolution style, α = 0.74 for retreat, and α = 0.73 for subordination. For the current sample, Cronbach’s alpha coefficient was α = 79 for positive conflict resolution styles, α = 0.79 for negative conflict resolution styles, α = 0.75 for retreat, and α = 0.72 for subordination.

### Relationship Assessment Scale (RAS)

The RAS [30] is 7 item self-report measure designed to determine relationship satisfaction. Respondents indicate on a scale from 1 (low) to 7 (high). Curun [31] tested the psychometric properties of RAS for a Turkish sample and found the reliability estimate for RAS as α = 0.86. The Cronbach’s alpha coefficient for the present example is α = 0.89.

### Need satisfaction in romantic relationship scale (NSRRS)

The NSRRS  [32] is 9 items self-report measure designed to determine need satisfaction in relationships. It consists of three subscales as ‘autonomy’, ‘competence’, and ‘relatedness’. Özdemir And Sagkal [33] tested the psychometric properties of RAS for a Turkish sample and found that a three-factor second-order hypothesized model in the Turkish sample showed an adequate fit to the data: χ 2 (23) = 72.121, p < .001, χ2 /df = 3.14, RMSEA = 0.07, 90% CI[0.05, 0.09], NFI = 0.90; CFI = 0.93, GFI = 0.97, AGFI = 0.94. Cronbach’s alpha coefficient for the total scale is α = 0.68 and 4-week interval test-retest reliability coefficients of the total scale is α = 0.78. For the current sample, Cronbach’s alpha coefficient was α = 78 for autonomy, α = 0.74 for relatedness, α = 0.69 for competence, and α = 0.87 for total scale.

### Data analyses

Data were analyzed in four steps using SPSS 22.0. Firstly, we investigated the properties of the variables. Secondly, we conducted a correlational analysis to test the relationships between ABRR, relationship satisfaction, need satisfaction, and conflict resolution skills. In the third step, the mediating role of ABRR in the relationships between conflict resolution styles and relationship satisfaction was tested using SPSS macro PROCESS (Model 4) [34]. Finally, the moderation role of need satisfaction in the relationships between ABRR and relationship satisfaction was tested (Model 1) [34]. The bias-adjusted confidence interval (CI) suggested by Hayes [34] was used in the study. The SPSS Macro Process program examines the total, direct, and indirect effect scores and the mediator and moderation variable’s possible effect on the dependent variable [35].

## Results

A correlational analysis indicated that ABRR was negatively correlated with relationship satisfaction, need satisfaction, and positive conflict resolution but positively correlated with negative conflict resolution, subordination, and retreat. Moreover, relationship satisfaction was positively correlated with positive conflict resolution and need satisfaction but negatively correlated with negative conflict resolution, subordination, and retreat (Table 1).

**Table 1:** Correlations and descriptive statistics (N = 333)

	1	2	3	4	5	6	7	8	9
1. ABRR	1	-,51**	-,23**	,37**	,20**	,32**	-,46**	-,33**	-,63**
2.Relationship Satisfaction		1	,16**	-,27**	-,19**	-,10*	,62**	,41**	,46**
3. Positive Conflict Resolution			1	-,29**	-,20**	-,15**	,23**	,21**	,30**
4. Negative Conflict Resolution				1	,15**	,09*	-,18**	-,18**	-,23**
5. Retreat					1	,34**	-,15**	-,05	-,20**
6. Subordination						1	-,08	-,10	-,26**
7. Relatedness							1	,54**	,44**
8. Competence								1	,41**
9. Autonomy									1
*X*	33,30	43,28	29,44	14,94	21,30	20,46	19,24	17,89	19,18
*Ss*	7,74	5,32	3,81	5,87	6,41	5,40	2,31	2,72	2,20
*Kurtosis*	1,17	-1,24	-0,17	0,83	-0,06	-0,11	-1,5	-1,04	-1,28
*Skewness*	0,92	1,23	-0,66	0,35	-0,60	-0,46	1,87	,71	,91

**p < .01 *p < .05

### Testing the Mediator Role of ABRR

**Fig. 1:**
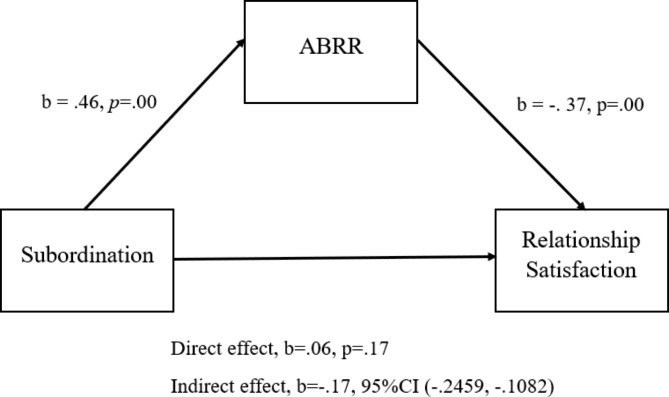
Mediation role of ABRR in the relationship between subordination and relationship satisfaction

We used Hayes’s [34] PROCESS macro (model 4) to test the mediator role of ABRR in the relationship between subordination, retreat, and relationship satisfaction. Analysis indicated that subordination significantly predicted ABRR (p < .001) and relationship satisfaction (p < .001). ABRR (p < .001) predicted relationship satisfaction. Moreover, subordination indirectly predicted (B = − 0.17, SE = 0.03, 95% [CI] = − 0.24, − 0.10) relationship satisfaction via ABRR (Fig. 1).

**Fig. 2:**
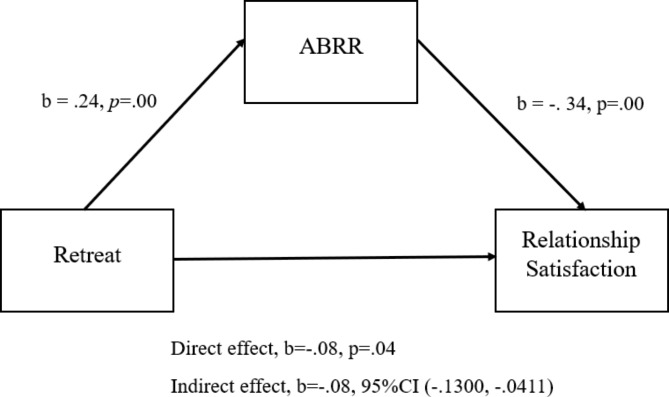
Mediation role of ABRR in the relationship between retreat and relationship satisfaction

Another result of the study that retreat significantly predicted ABRR (p < .001) and relationship satisfaction (p < .001). ABRR (p < .001) predicted relationship satisfaction. Moreover, retreat indirectly predicted (B = − 0.08, SE = 0.02, 95% [CI] = − 0.13, − 0.03) relationship satisfaction via ABRR (Fig. 2).

### Testing the moderation role of need satisfaction

**Fig. 3:**
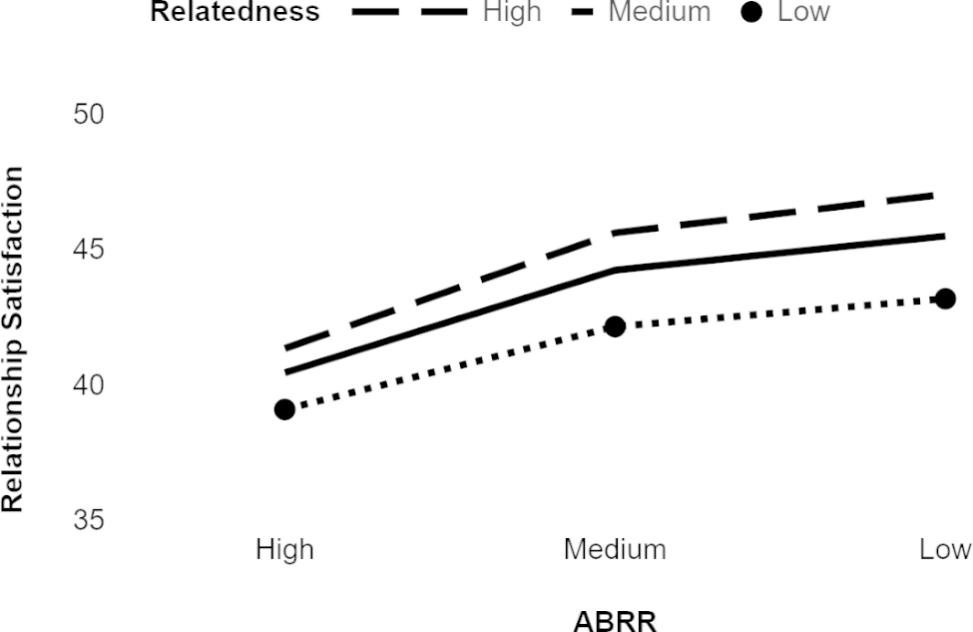
Moderation role of relatedness in the relationship between ABRR and relationship satisfaction

We used Hayes’s [34] PROCESS macro (Model 1) to test the moderation role of relatedness in the relationships between ABRR and relationship satisfaction. The results revealed that relatedness (p < .001) and ABRR (p < .001) predicted relationship satisfaction, and the interaction effect of ABRR and relatedness was significant as well (ΔR^2^ = 0.46, p = .021). Simple slope analysis revealed that the relationship between ABRR and relationship satisfaction is significant when levels of relatedness are low (b = − 0.15, p = .00) and high (b = − 0.25, p < .00), but this relation is strong when the levels of relatedness are high (Table 2; Fig. 3).

**Table 2:** Moderation Role of Relatedness

		Outcome: Relationship Satisfaction			
Predictor Variables	*B*	*SE*	*t*	*Model R* ^*2*^	
ABRR	,30	,22	1,39	,46**	
Relatedness ABRR x Relatedness	2,13 -,02	,44 ,01	4,75** -2,31*		

**p < .01 *p < .05

**Fig. 4:**
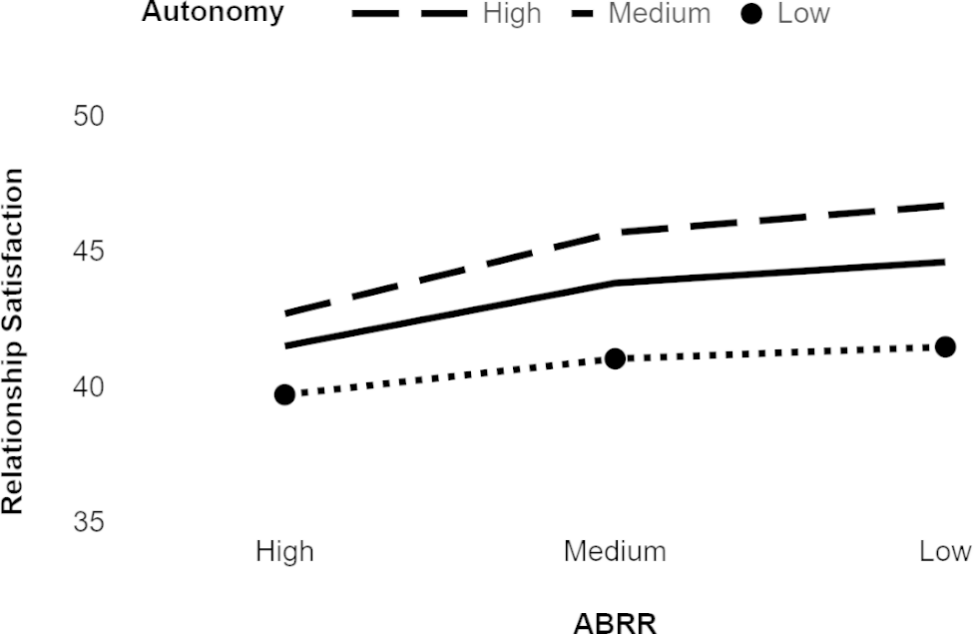
Moderation role of autonomy in the relationship between ABRR and relationship satisfaction

Another result of the study was that autonomy (p < .001) and ABRR (p < .001) predicted relationship satisfaction, and the interaction effect of ABRR and autonomy was significant as well (ΔR^2^ = 0.31, p = .00). Simple slope analysis revealed that the relationship between ABRR and relationship satisfaction is significant when levels of autonomy are low (b = − 0.19, p = .00) and high (b = − 0.34, p < .00), but this relation is strong when the levels of autonomy are high (Table 3; Fig. 4).

**Table 3:** Moderation Role of Autonomy

		Outcome: Relationship Satisfaction			
Predictor Variables	*B*	*SE*	*t*	*Model R* ^*2*^	
ABRR	,43	,25	1,74	,46**	
Autonomy ABRR x Autonomy	1,97 -,03	,53 ,01	3,72** -2,80**		

**p < .01 *p < .05

## Discussion

This study was designed to analyze the mediator role of ABRR in the relationship between conflict resolution styles(subordination and retreat) and relationship satisfaction and the moderation role of need satisfaction(relatedness and autonomy) in the relationship between ABRR and relationship satisfaction.

### Mediator role of ABRR

According to the findings, ABRR has a mediator role in the relationship between subordination and retreat with relationship satisfaction. In the existing literature, it is observed that ABRR has an effect on subordination and retreat [36] and relationship satisfaction [17, 19]; subordination and retreat have an effect on relationship satisfaction [11, 13]. Therefore, the present findings are consistent with the findings of our study. Another study stated that destructive conflict resolution styles might cause adolescents and emerging adults to experience unsatisfactory relationships and increased aggression [37]. Indeed, the findings of this study suggest that retreat and subordination have a positive effect on ABRR, which may lead to a decrease in relationship satisfaction. Considering that retreat and subordination may be among destructive conflict resolution styles, the findings of this study support [37]. In other words, individuals who use subordination and retreat conflict resolution styles may be more likely to experience ABRR. Thus, the increased probability of experiencing ABRR may alleviate the effect of subordination and retreat on relationship satisfaction. Based on this finding, examining the existence of possible ABRR while evaluating the relationship satisfaction of individuals using the subordination and retreat conflict resolution style is recommended.

### Moderation role of autonomy and relatedness

Research findings show that autonomy and relatedness have a moderation role in the relationship between ABRR and relationship satisfaction. According to a meta-analysis study, partners whose needs of competence, relatedness, and autonomy are met, it is seen that they; experience more positive emotions, increased self-esteem, high levels of relationship satisfaction and relationship commitment, perceive less conflict, and are less defensive in conflict [24]. La Guardia [38] concluded that individuals with high need satisfaction in romantic relationships have increased levels of emotional awareness and are more open to their partners emotionally. Another possible reason for autonomy and relatedness to have a moderation role in this study is that the participants were emerging Turkish adults. According to a study by Kagıtçıbası [28], it is seen that individuals in Turkey have an autonomous-related self-structure. One study revealed that the coexistence of autonomy and relatedness affects relationship satisfaction [39]. Additionally, married individuals who emphasize autonomy and relatedness have the highest levels of self-validation [40]. As a result, it can be said that the autonomous-related self-characteristics of Turkish culture may affect the moderation role of autonomy and relatedness. Finally, in the current study, it is seen that the needs of relatedness and autonomy have a buffering role in the relationship between ABRR and relationship satisfaction. In other words, individuals aware of their needs experience relationship satisfaction even if exposed to abuse.

### Limitations and future directions

The findings of the current study should be evaluated within the following limitations. The most important limitation of the study is that it is cross-sectional. Although the findings indicated a mediator role of abusive behavior in romantic relationships and moderation roles of relatedness and autonomy, the cross-sectional design does not allow us to evaluate the relationships between the variables within the framework of cause-effect relationships. In order to confirm the mediator roles of ABRR and moderation roles of relatedness and autonomy, further studies should be conducted in a longitudinal manner.

Another limitation of the study is the use of the convenience sampling method. The convenience sampling method has limitations in terms of the representativeness of the population.

Another limitation may be that this study was conducted in the context of romantic relationships in Turkish culture. Therefore, repeating this study within different cultures may affect the literature regarding the generalizability of the findings.

Another limitation of the present study is that the sample consists of more women than men; this may limit the generalizability of the findings to both genders. Therefore, repeating this study with equal numbers of participants from both genders may contribute to the literature regarding the generalizability of the findings. In addition, in this study, most participants were young women, and scales for subjective evaluation and statements were used. Considering that “need satisfaction” and “relationship satisfaction” among the variables examined depend on the participant’s mood, young women may evaluate the scales quite differently at different stages of the menstrual cycle. Therefore, it is recommended for future studies to collect data on the regularity of the menstrual cycle of female participants.

Another limitation of the study may be the participants’ developmental period and marital status. The research was solely conducted with emerging adults in romantic relationships, so it is recommended for future studies to work with married people or with different developmental periods and to collect data on whether there is differentiation.

Another limitation may be the inconsistency in research findings on the relationship between ABRR and relationship satisfaction. One possible reason for this inconsistency may be a trauma history. Individuals with a history of trauma, such as abusive, neglectful, or seductive behaviours in their family of origin, may be prone to repeat similar patterns in their current romantic relationships [41] For this reason, it is recommended that the trauma history should also be evaluated in future studies. Therefore, it is also recommended to evaluate trauma history in future studies.

## Conclusions

Our results showed that subordination and retreat were significant predictors of relationship satisfaction. Mediation analyses revealed that ABRR has a mediator role in this relationship. In other words, ABRR mediated the indirect effect of subordination and retreat on relationship satisfaction. Another result of the research showed that relatedness and autonomy have a moderator role in the relationship between ABRR and relationship satisfaction. In other words, the negative effect of ABRR on relationship satisfaction was lower in individuals with high relatedness and autonomy than in those with low relatedness and autonomy. The results may have implications for future studies on relationship satisfaction, for the development of intervention programs and group work on relationship satisfaction, and for therapists working with couples.

## Data Availability

The data that support the findings during the current study are available from the corresponding author upon reasonable request.

## List of abbreviations

ABRR: Abusive behaviour in romantic relationship
ABS: Abusive Behaviour Scale For Romantic Relationship
CRSS: Conflict Resolution Styles Scale for Romantic Relationships
RAS: Relationship Assessment Scale
NSRRS: Need Satisfaction in Romantic Relationship Scale
