# Correction: abusive behaviours in relationships, need satisfaction, conflict styles and relationship satisfaction: mediation and moderation roles

**DOI:** 10.1186/s40359-023-01222-2

**Published:** 2023-06-19

**Authors:** Ahu Aricioglu, Sefa Kaya

**Affiliations:** 1 Psychological Counseling and Guidance Department, Pamukkale University, Denizli, Turkey; 2 Psychological Counseling and Guidance Department, Pamukkale University, 359 Sokak no:60 Daire:15 Buca, Denizli, Izmir, Turkey

**BMC Psychology (2023) 11:160.**

10.1186/s40359-023-01202-6.

Following publication of the original article, it was brought to the journal’s attention that the following had been erroneously incorporated into the author list: ‘Orcid Number of & Orcid Number’. The author list has since been corrected. The publisher thanks you for reading and apologizes for any inconvenience caused by this formatting error.

